# The effect of physical barriers under a raised house on mosquito entry: an experimental study in rural Gambia

**DOI:** 10.1186/s12936-024-04889-z

**Published:** 2024-04-08

**Authors:** Majo Carrasco-Tenezaca, Musa Jawara, John Bradley, Umberto D’Alessandro, David Jeffries, Jakob B. Knudsen, Steve W. Lindsay

**Affiliations:** 1 Department of Biosciences, Durham University, Durham, UK; 2 Centro de Investigación Para la Salud en América Latina, Pontificia Universidad Católica del Ecuador, Quito, Ecuador; 3 Medical Research Council Unit The Gambia at the London, School of Hygiene and Tropical Medicine, Banjul, The Gambia; 4 London School of Hygiene & Tropical Medicine, London, UK; 5 Royal Danish Academy-Architecture, Design and Conservation, Copenhagen, Denmark

**Keywords:** *Anopheles gambiae*, Housing, Malaria, Mosquitoes, Sub-Saharan Africa

## Abstract

**Background:**

*Anopheles gambiae*, the major malaria mosquito in sub-Saharan Africa, feed largely indoors at night. Raising a house off the ground with no barriers underneath reduces mosquito-house entry. This experiment tested whether walling off the space under an elevated hut affects mosquito-hut entry.

**Methods:**

Four inhabited experimental huts, each of which could be moved up and down, were used in rural Gambia. Nightly collections of mosquitoes were made using light traps and temperature and carbon dioxide levels monitored indoors and outdoors using loggers. Each night, a reference hut was kept at ground level and three huts raised 2 m above the ground; with the space under the hut left open, walled with air-permeable walls or solid walls. Treatments were rotated every four nights using a randomized block design. The experiment was conducted for 32 nights. Primary measurements were mosquito numbers and indoor temperature in each hut.

**Results:**

A total of 1,259 female *Anopheles gambiae *sensu lato were collected in the hut at ground level, 655 in the hut with an open ground floor, 981 in the hut with air-permeable walls underneath and 873 in the hut with solid walls underneath. Multivariate analysis, adjusting for confounders, showed that a raised hut open underneath had 53% fewer mosquitoes (95% CI 47–58%), those with air-permeable walls underneath 24% fewer (95% CI 9–36%) and huts with solid walls underneath 31% fewer (95% CI 24–37%) compared with a hut on the ground. Similar results were found for *Mansonia* spp. and total number of female mosquitoes, but not for *Culex* mosquitoes where hut entry was unaffected by height or barriers. Indoor temperature and carbon dioxide levels were similar in all huts.

**Conclusion:**

Raising a house 2 m from the ground reduces the entry of *An. gambiae* and *Mansonia* mosquitoes, but not *Culex* species. The protective effect of height is reduced if the space underneath the hut is walled off.

**Supplementary Information:**

The online version contains supplementary material available at 10.1186/s12936-024-04889-z.

## Background

By 2050, the population of sub-Saharan Africa is projected to increase by 1.05 billion, an increase roughly representing the present population of India or China, becoming the most populated region on the world around 2060 [1]. Most of the projected rise in population will occur in secondary cities and towns, with urban areas growing in population by 87% [1]. Whilst settlements will expand in area, land scarcity and rising land prices require new solutions to provide affordable high-density homes for the increasing population. Inevitably, this means constructing multi-storey housing [2, 3].

Although major reductions in malaria have been recorded since the turn of the century, the disease remains a major public health problem in the African region with 234 million cases in 2021 [4]. About 79% of these cases were infected with malaria parasites indoors at night [5], highlighting the importance of protecting people from malaria mosquitoes in their homes. Since 2000, the major interventions used for protecting people indoors in the region have been insecticide-treated nets (ITN), and, to a lesser extent, indoor residual spraying [4]. Today malaria control has stalled, with the number of cases increasing in some countries. Complementary strategies are, therefore, needed to prevent a resurgence of malaria, particularly during the COVID-19 pandemic where malaria control is no longer the public health priority and service disruptions are common.

Improved housing could be used as a complementary strategy to reduce the force of malaria infection [6]. Changes to the structure of a house can directly and indirectly reduce malaria mosquito house entry. Mosquito screens on doors and windows act as physical barriers to mosquito ingress [6] and, by increasing ventilation, reduce the number of mosquitoes that can locate entry points in a house [7, 8]. Good ventilation reduces the concentration of carbon dioxide indoors, an important gas used by malaria mosquitoes for locating a host [9]. Incorporating two screened windows into a single-roomed house reduces the entry of *Anopheles gambiae*, the principal African malaria vector, by 79% [8]. Improved ventilation can also have the additional beneficial effect of cooling the house at night, making it more likely that people will sleep under an ITN, since having a ‘hot house’ is one of the major reasons why people will not use a net at night [10].

Raising experimental huts off the ground reduces the number of malaria mosquitoes entering the huts [11], with huts at 2 m above the ground having 68% fewer mosquitoes than those on the ground. Since *An. gambiae* are low-flying mosquitoes, with most flying no more than one metre above the ground [12, 13], it was hypothesized that these mosquitoes find it difficult to locate the carbon dioxide, and probably other attractants, emanating from the raised huts. In these experiments the huts were supported by four columns at each corner, with no walls under the huts. In the real-world, many houses constructed off the ground have the ground floor walled to create an extra room or to store objects and food. In the 1970s, Gillies and Wilkes, in the same study location as the present study, showed that *An. gambiae* can fly over a six metre high fence [14], suggesting that the protection resulting from raising a house off the ground would be reduced or nullified if there were physical barriers under the hut, perhaps causing low-flying mosquitoes to fly upwards, increasing the numbers entering the hut.

There were two primary objectives of this study, to: (1) determine whether mosquito-hut entry was affected when the space directly beneath the hut was enclosed with different types of walls and (2) find out whether these alterations affected indoor temperature and carbon dioxide levels in elevated huts.

## Methods

### Study area

The study took place in Wellingara village (N 13° 33″ 365′, W 14° 55″ 461′), Central River Region, The Gambia. The study site was located on the edge of the village close to a large area of irrigated rice. The study took place during the rainy season 2021, from August 23rd to October 4th, when *An. gambiae *sensu lato (*s.l*.) are common [14].

### Experimental huts

**Fig. 1:**
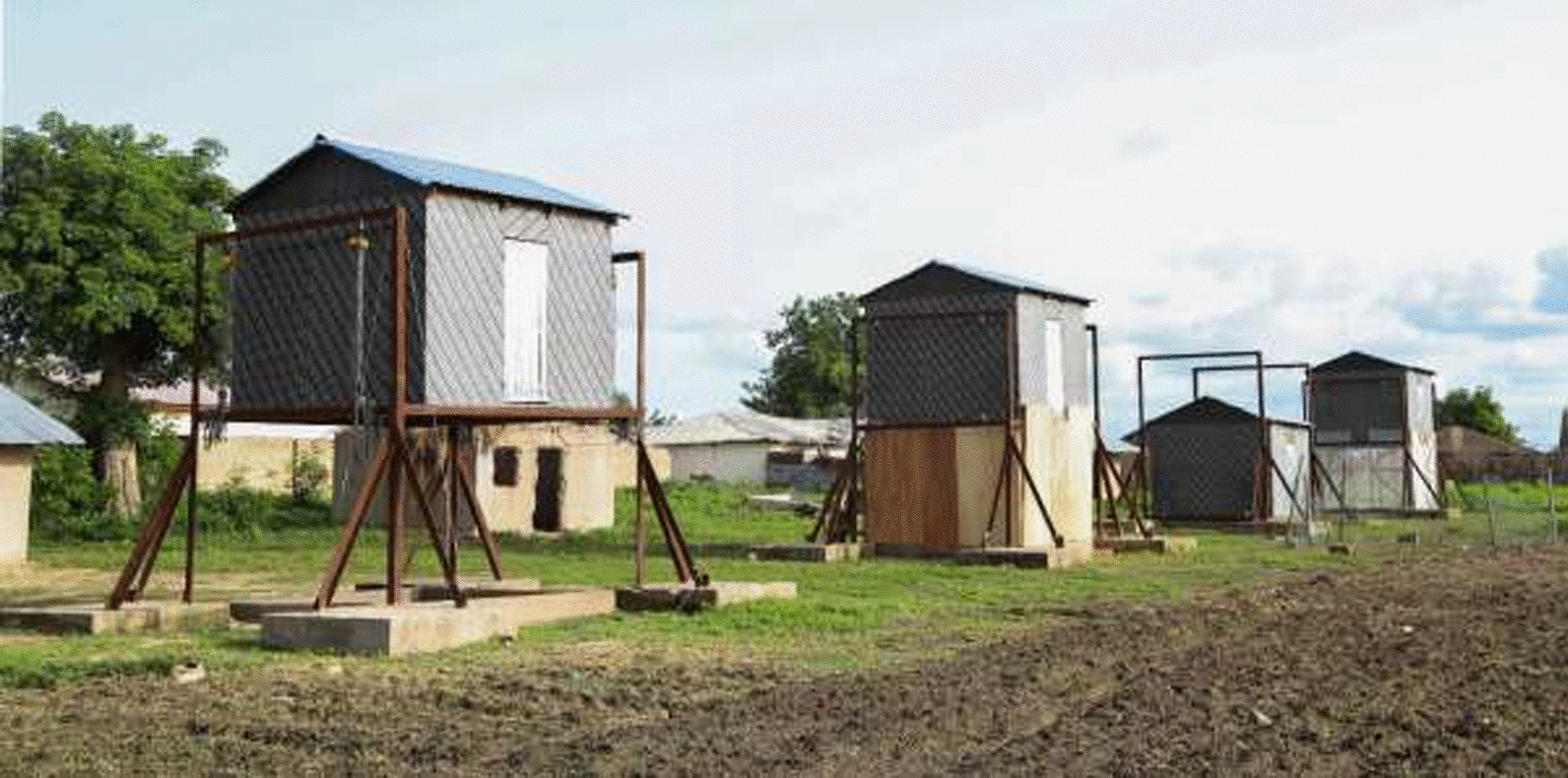
Experimental huts. From left to right, raised hut without walls below the hut, raised hut with solid wooden walls underneath, hut at ground level (control), raised hut with air-permeable walls underneath. Stairs are shown on the rear face of the second hut from the left only (although in practise all elevated huts had stairs)

As described previously [11], four experimental huts were built, 10 m apart, along a straight line on the western edge of the village and close to an irrigated rice field (Fig. 1). The huts were designed to resemble single-room houses common in rural Gambia, although they were smaller and constructed from lightweight materials to make it easier and safer to raise and lower them. All sides of each hut were 3.1 m wide and 2.2 m high with two doors on opposite sides (east and west) and no windows. The only mosquito entry points were through 20 mm × 700 mm wide gaps that extended along the length of the top and bottom of each door. These gaps simulated badly-fitting doors common in the local area. For this study, the floor of each hut was positioned at one of two heights, the reference hut at 0 m and the comparator huts at 2 m from the top of the concrete plinths. In order that the huts could be moved up and down, they were supported by two steel frames embedded in four concrete plinths on the ground, one at each corner. Because of this design, when a hut was on the ground, there was a space directly under the hut floor.

Each hut had two bamboo beds positioned next to the walls in an east–west direction, leaving a clear space between the doors (Additional file 1: Fig. S1). Each raised hut had a fixed wooden staircase on the east side, away from the rice field, the major source of mosquitoes (Additional file 1: Fig. S2). The staircase was left in place on nights a hut was raised high above the ground to facilitate safe entry and exiting.

**Fig. 2:**
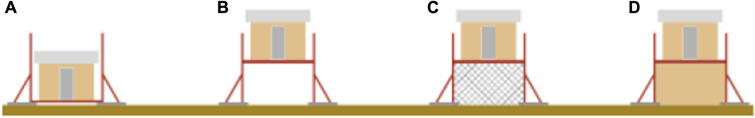
Hut typologies used in the experiment. **A** control hut at 0 m, **B** hut at 2 m with open ground storey, **C** hut at 2 m with air-permeable walls on the ground storey and (**D**) hut at 2 m with solid walls on the ground storey

For each experimental session, the huts were arranged into four typologies: (1) the reference hut on the ground, and three huts raised 2 m above the ground, with (2) open space underneath the hut, (3) air-permeable walls on the ground floor and (4) solid walls on the ground floor (Figs. 1, 2). The air-permeable enclosure was constructed using untreated fly screen walls (white plastic, 708 × 630 holes per sq m, Vestergaard-Frandsen group, Kolding, Denmark) to surround the ground storey (the space immediately beneath the hut). The solid enclosure was made from 12 mm thick plywood boards.

### Study design

This experiment was designed primarily to determine whether adding solid or air-permeable walls under an elevated hut affected mosquito-hut entry and indoor temperature. Four identical experimental huts were constructed, each of which could be raised and lowered in a frame. For each block, three huts were raised 2 m above the ground and one kept at ground level. Entry of mosquitoes into each hut was only possible through narrow gaps at the top and bottom of both doors. Treatments were changed every four nights, following a replicated Latin rectangle design (Additional file 1: Table S1). The experiment was conducted for nine blocks of four nights each.

### Human subjects

A village meeting was organized to explain the study to the Alkalo (village leader) and the village elders and to request their approval for the study. Following the approval of the elders, another meeting was conducted with the villagers to explain the purpose of the study, gain their approval and recruit volunteers. Both meetings were conducted in Mandinka, the local language. Only healthy men, over 18 years old, resident in Wellingara village, who provided signed-witnessed consent were recruited to the study. Participants were randomly assigned to a pair at the beginning of the study. However, two middle-aged men, for cultural reasons, did not want to sleep in the same room as younger men, so were paired together for nights 5–36. This pseudo-randomized pairing was maintained for the remainder of the study. Each night, each pair of men slept in each hut under individual ITNs (Olyset Net, 1.3 m wide, 1.5 m high and 1.8 m in length, Sumitomo Chemicals, Japan), from 21.00 h to 07.00 h the following morning (Supplementary Fig. 1) with their heads pointing west, towards the rice fields. Each pair of sleepers were rotated between huts each night so that at the end of each session’s block, each pair had slept in each hut. Two field assistants were stationed on site each night in a separate room to supervise the study subjects during the night and to record study data.

### Outcomes

The main entomological outcome was the mean number of female *An. gambiae* s.l. collected in light traps and the main environmental outcomes were mean temperature of huts and mean indoor carbon dioxide levels before midnight, the time when most people go to bed and decide whether to use a bed net or not. Primary mosquito collection was done using one light trap (CDC-Centers for Disease Control and Prevention, Miniature light trap model 512, US John W. Hock Ltd, Gainsville, USA) per hut, which ran when the men were in the huts, from 21.00 h to 07.00 h the following morning. Each light trap was located between the foot ends of the two beds, with the light 1 m above the floor. Secondary mosquito collections of living and dead mosquitoes were performed by trained field assistants every morning, using a Prokopack aspirator (Model 1419, John W. Hock Ltd, Gainsville, USA) for 10 min in each hut. Every experimental night, the field assistants conducted two supervisory visits, at 00.00 h and 06.00 h, to ensure that men were in the huts, light traps were working properly and to assess bed net use. Each morning, collected mosquitoes were killed in a − 20 °C freezer. Mosquitoes were identified using standard morphological identification keys [15, 16], and a selection of female *An. gambiae* identified to species by PCR analysis [17–19].

Environmental conditions were recorded from 21.00 h to 07.00 h and analysed in two separate sections: before midnight, when people are typically going in and out of their houses, and after midnight, when people are, mainly, indoors sleeping [20]. Indoor temperature and relative humidity were measured in each hut every 30 min using one data logger (TGU 4500, Tinytag, UK) located in the centre of the room and 1 m above the floor. Carbon dioxide concentration was recorded every 30 s with a data logger (1% CO_2_ + Rh/T Data Logger GasLab, Florida, USA) located between in the middle of the hut near the head of the bed, 1.2 m above the floor according to the manufacturer’s recommendation (Additional file 1: Fig. S1). Outdoor temperature, relative humidity, wind speed, wind direction and precipitation were recorded every 30 min using an automatic weather station (MiniMet, Skye Instruments, Llandrindod Wells, UK), located 10 m from the line of the huts, in the middle of the row of huts.

### Data analyses

The analysis used IBM SPSS Statistics 27 and Stata version 17. The sample size was estimated using a computer simulation based on data from a study conducted in the same area in 2017 [7], in which the mean number of *An. gambiae s.l.* collected indoors over 25 nights was 6.4 mosquitoes/hut/night (SD 7.1) and supported by a study conducted in 2019 in the same location and huts [8], where the mean number of *An. gambiae s.l.* collected indoors over 40 nights was 53/hut/night (SD 56). The present study was thus powered to detect an intervention that reduces the number of mosquitoes found indoors by at least 75% at the 5% level of significance and 90% power. In the simulation, the 4 × 4 Latin square was repeated from three to 10 times (i.e. 12–40 nights). The simulation showed that eight, 4 × 4 Latin squares would provide sufficient power to detect a 75% reduction in mosquito hut entry (i.e. 32 nights of collections). Here, the results of nights 5 to 36 (n = 32 nights) are reported since nights 1–4 collected few mosquitoes and the composition of the pairs remained unchanged from nights 5 to 36.

To assess the effect of ground floor treatments to mosquito hut entry and indoor climate a generalized estimating equation using a negative binomial model with a log link function was used for mosquito count data and a normal distribution with identity link for temperature and carbon dioxide, since they were continuous variables and normally distributed. In addition to hut height, hut treatment, hut position, sleeper pair and number of nights in the model were included as fixed effects. To examine the relationship between carbon dioxide concentration and covariates, linear regression was used. Polar plots were used to depict the direction and strength of the wind during the day and night. A linear regression model was used to examine the relationship between female *An. gambiae* and *Mansonia* spp.

## Results

### Mosquito collections

**Fig. 3:**
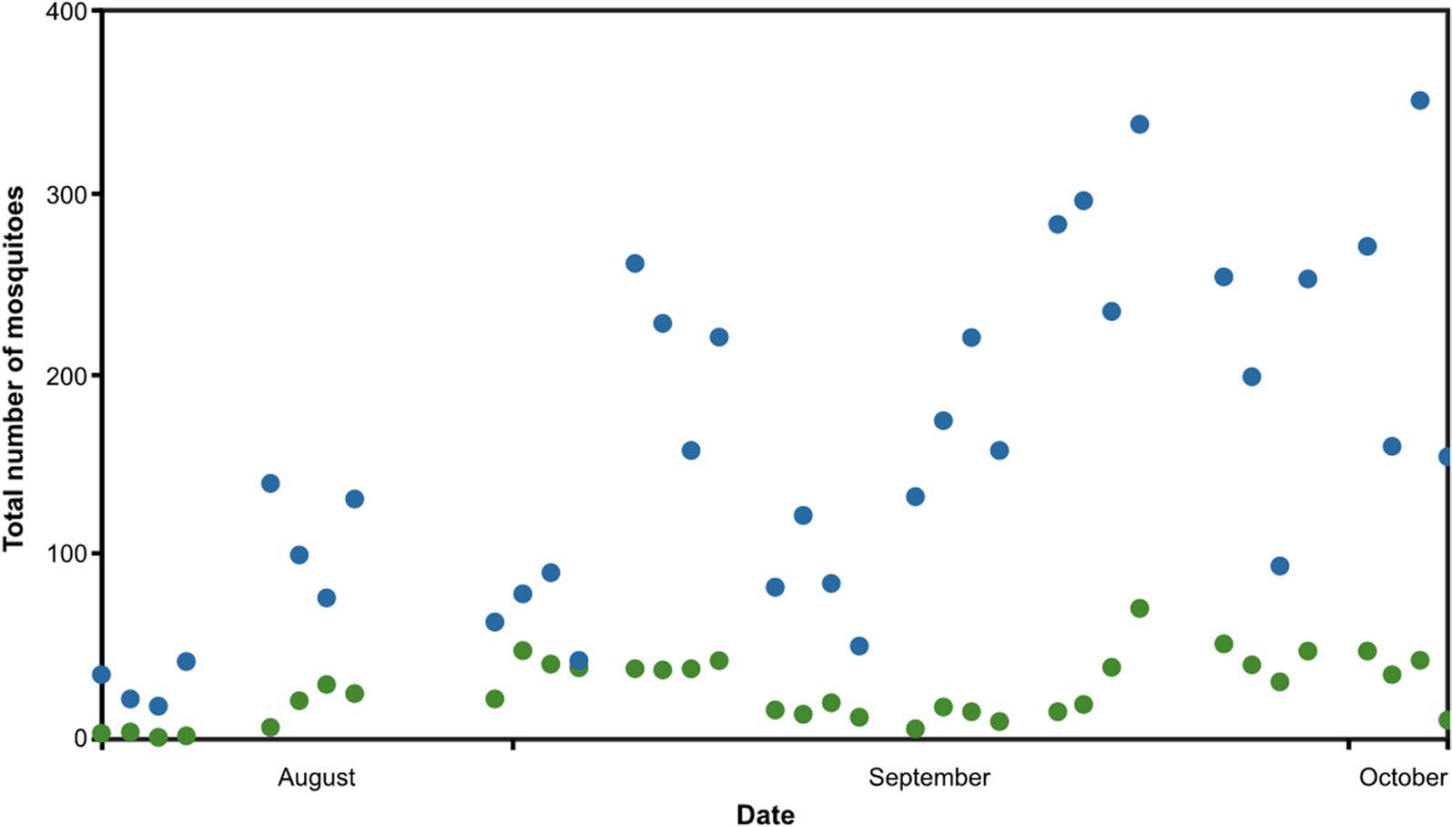
Relative abundance of *An. gambiae s.l.* and *Mansonia* spp collected each night during the study. Where green dots = *An. gambiae* and blue dots = *Mansonia* spp

A total of 28,629 male and female mosquitoes were collected in the experimental huts using light traps over 32 nights. Of these, 3768 (13%) were female *An. gambiae s.l.*, 21,982 (77%) female *Mansonia* spp, 2441 (9%) female *Culex* spp. and the rest were other male and female anophelines and *Aedes aegypti* (Additional file 1: Table S2). PCR analysis identified female members of the *An. gambiae* complex as *Anopheles coluzzii* (77.5%, 93/120), *Anopheles arabiensis* (20%, 20/120), and *An. gambiae *sensu stricto (2.5%, 3/120). Mosquito numbers were low during the first week of the study but rose towards the end of August with large variations from week to week (Fig. 3). Numbers of *Mansonia* spp. increased during the study and were consistently greater each night than the number of female *An. gambiae*.

There was a linear relationship between the total numbers of female *An. gambiae* and *Mansonia* spp. captured in each hut each night (adjusted R^2^ = 0.401, df = 142, p ≤ 0.001). This relationship was also found in the house on the ground (adjusted R^2^ = 0.559, df = 34, p ≤ 0.001), in elevated houses with an open ground storey (adjusted R^2^ = 0.326, df = 34, p ≤ 0.001), air-permeable walls on the ground storey (adjusted R^2^ = 0.190, df = 34, p = 0.005) and solid walls on the ground (adjusted R^2^ = 0.332, df = 34, p ≤ 0.001).

**Fig. 4:**
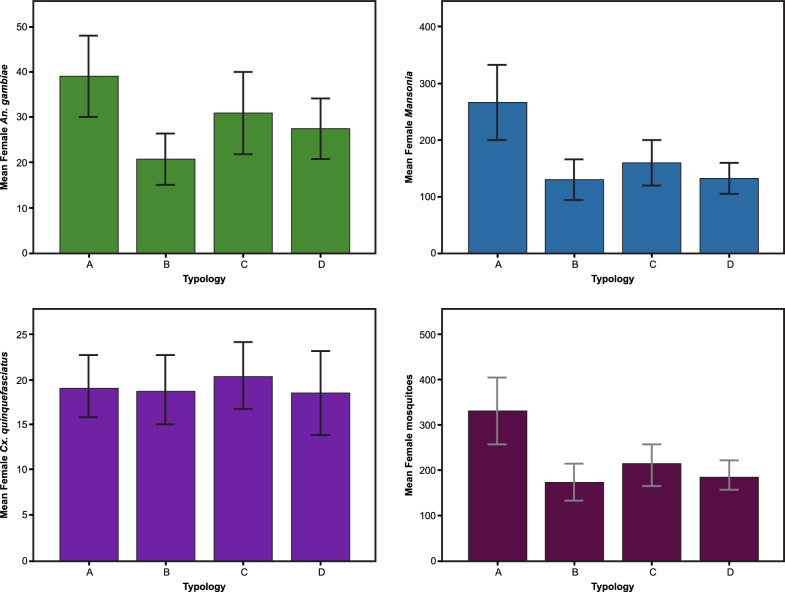
Mean mosquito house entry in different hut typologies.** A** Hut on the ground (control). **B** Raised hut with open ground storey.** C** Raised hut with air-permeable walls on the ground storey, and** D** raised hut with solid wall on the ground storey. Error bars are 95% confidence intervals. NB scales differ for each mosquito group. Green bars represent female An.* gambiae* s.l., blue bars female *Mansonia*, purple bars female Cx. *quinquefasciatus* and magenta bars are all female mosquitoes.

Mean nightly indoor density of female *An. gambiae s.l.* was 39 (95% CI 30–48) in the hut on the ground, 20 (95% CI 15–26) in the raised hut with an open ground storey, 31 (95% CI 21–40) in the hut with air-permeable walls and 27 (95% CI 21–34) in the hut with solid walls (Fig. 4).

**Fig. 5:**
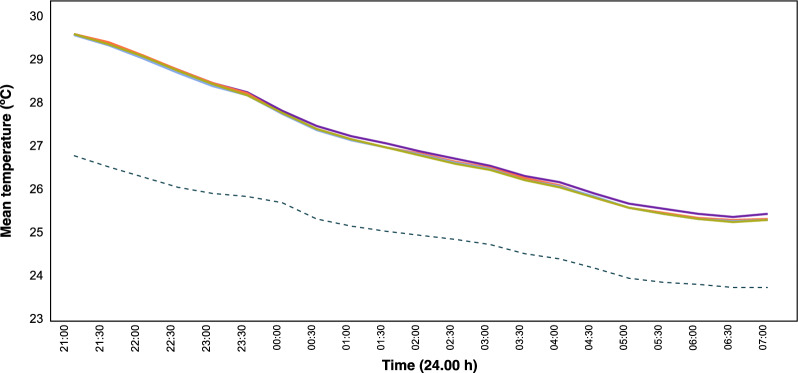
Mean outdoor and indoor temperatures during the night. Where purple line = hut on the ground (control); green line = raised hut with open ground storey; light blue line = raised hut with air-permeable walls on the ground storey; orange line = raised hut with solid wall on the ground storey and dashed line = outdoor values

The adjusted analysis, allowing for nights, sleeper pair and hut position, showed that the number of female *An. gambiae* entering the elevated huts was reduced in all elevated huts compared to the hut on the ground. There were 53% (95% CI 47–58%) fewer female *An. gambiae* in the hut at 2 m with an open ground storey, 24% fewer (95% CI 9–36%) in the hut at 2 m with air-permeable walls on the ground storey and 31% (95% CI 24–37%) fewer in the hut at 2 m with solid walls on the ground storey, compared to the hut at 0 m (Table 1). Similar results were seen with *Mansonia* spp. and total female mosquitoes, but not *Culex* spp. A second adjusted analysis only including the elevated huts at 2 m and using the hut with open ground storey as reference. Here there were 63% (95% CI 36–96%) more female *An. gambiae* in the hut with air-permeable walls on the ground storey and 45% (95% CI 36–56%) more in the hut with solid walls compared to the raised hut with an open ground storey. Similar findings were found for for *Mansonia* spp. and total female mosquitoes, but not *Culex* spp.

**Table 1:** Female mosquitoes collected at different heights and adjusted analysis for covariates

Hut typology	Total (N)	Mean no./hut/night	Mean ratio (95% CI)	Effect estimate (95% CI)	*P*	Mean ratio (95% CI)	Effect estimate (95% CI)	*P*
*Anopheles gambiae*								
Hut on the ground	1259	35	Reference					
Raised hut with open ground storey	655	18	0.47 (0.42–0.53)	− 53% (− 47 to − 58)	< 0.001	Reference		
Raised hut with air-permeable walls on the ground storey	981	27	0.76 (0.64–0.91)	− 24% (− 9 to − 36)	0.002	1.63 (1.36 to 1.96)	63% (36 to 96)	< 0.001
Raised hut with solid walls on the ground storey	873	24	0.69 (0.63–0.76)	− 31% (− 24 to − 37)	< 0.001	1.45 (1.36 to 1.56)	45% (36 to 56)	< 0.001
*Mansonia* spp.								
Hut on the ground	8535	240	Reference					
Raised hut with open ground storey	4145	120	0.48 (0.41–0.57)	− 52% (− 43 to − 59)	< 0.001	Reference		
Raised hut with air-permeable walls on the ground storey	5021	142	0.62 (0.54–0.72)	− 38% (− 28 to − 46)	< 0.001	1.31 (1.27 to 1.36)	31% (27 to 36)	< 0.001
Raised hut with solid walls on the ground storey	4281	121	0.54 (0.49–0.59)	− 46% (− 41 to − 51)	< 0.001	1.14 (1.07 to 1.21)	14% (7 to 21)	< 0.001
*Culex* spp.								
Hut on the ground	610	18	Reference					
Raised hut with open ground storey	598	17	0.92 (0.83–1.03)	− 8% (− 17 to 3)	0.15	Reference		
Raised hut with air-permeable walls on the ground storey	645	19	1.06 (0.87–1.30)	6% (− 13 to 30)	0.55	1.13 (0.95 to 1.36)	13% (− 5 to 36)	0.169
Raised hut with solid walls on the ground storey	588	17	0.89 (0.85–0.93)	− 11% (− 7 to − 15)	< 0.001	0.34 (0.93 to 0.80)	− 66% (− 20 to − 7)	0.343
*All female mosquitoes*								
Hut on the ground	10,478	295	Reference					
Raised hut with open ground storey	5451	157	0.52 (0.45–0.59)	− 48% (− 41 to − 55)	< 0.001	Reference		< 0.001
Raised hut with air-permeable walls on the ground storey	6715	190	0.69 (0.60–0.79)	− 31% (− 21 to − 40)	< 0.001	1.34 (1.33 to 1.35)	34% (33 to 35)	< 0.001
Raised hut with solid walls on the ground storey	5814	165	0.59 (0.55–0.64)	− 41% (− 36 to − 45)	< 0.001	1.15 (1.10 to 1.19)	15% (10 to 19)	< 0.001

General linearised modelling results, adjusted for hut position, sleeper pair and night

*CI* confidence intervals

### Temperature recordings

Huts were consistently ~ 3 ºC warmer than outside (Fig. 5). Indoor temperature declined steadily during the night from a mean 29.5 ºC at 21.00 h to a mean of 25.4 ºC at 07.00 h. Multivariate analysis, adjusting for confounders, showed a borderline significant reduction of 6% (95% CI 0–13%) in indoor temperature during the first part of the night in a raised hut with air-permeable walls in the ground storey compared with the hut at ground level (Table 2). During the second part of the night the raised hut with solid walls on ground storey had a 7% (95% CI 0–14%) lower temperature compared with the hut on the ground. Wind was predominantly from the north-west and mean wind speed was 0.50 ms^−1^ (95% CI 0.45–0.63) from 20.00–23.59 h and 0.58 ms^−1^ (95% CI 0.56–0.67) from 00.00–06.59 h (Additional file 1: Fig. S2).

**Fig. 6:**
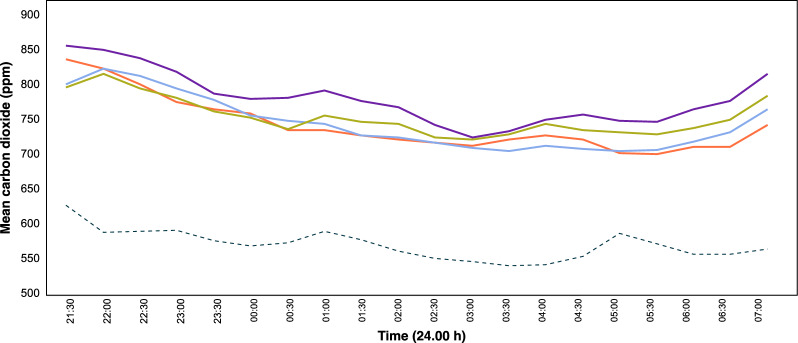
Mean carbon dioxide from 21.00 h to 07.00 h in different house typologies. Where purple line = hut on the ground (control); green line = raised hut with open ground storey; light blue line = raised hut with air-permeable walls on the ground storey; orange line = raised hut with solid walls on the ground storey and dashed line = outdoor values

**Table 2:** Temperature and carbon dioxide measurements before and after midnight

Postion of logger	Temperature					
	21.00 h to 23.30 h			00.00 h to 07.00 h		
	Average (ºC)	Difference from control house	*P*	Average (ºC)	Difference from control house	*P*
Outdoors	26.2 (25.6–26.9)			26.2 (25.6–26.9)		
Hut on the ground	28.9 (28.1–29.7)	Reference		26.6 (26.1–27.1)	Reference	
Raised hut with open ground storey	28.9 (28.1–29.7)	0.98 (0.83–1.16)	0.832	26.5 (25.9–27.0)	0.90 (0.75–1.07)	0.222
Raised hut with air-permeable walls on the ground storey	28.8 (28.1–29.6)	0.94 (0.87–1.00)	0.061	26.5 (26.0–27.0)	0.91 (0.81–1.03)	0.138
Raised hut with solid walls on the ground storey	28.9 (28.1–29.7)	1.00 (0.95–1.06)	0.896	26.5 (26.0–27.1)	0.93 (0.86–1.00)	1.180

Postion of logger	Carbon dioxide					
	Average from 21.00 h to 23.30 h			Average from 00.00 h to 07.00 h		
	Average (ppm)	Difference from control house	*P* value	Average (ppm)	Difference from control house	*P* value
Outdoors	598 (548–648)			570 (537–602)		
Hut on the ground	836 (782–891)	Reference		766 (725–806)	Reference	
Raised hut with open ground storey	788 (736–840)	0.65 (0.40 to 1.08)	0.095	738 (704–771)	0.81 (0.73 to 0.89)	< 0.001
Raised hut with air-permeable walls on the ground storey	798 (753–843)	0.53 (0.43 to 0.66)	< 0.001	727 (693–761)	0.58 (0.44 to 0.77)	< 0.001
Raised hut with solid walls on the ground storey	801 (758–844)	0.70 (0.45 to 1.11)	0.127	725 (694–755)	0.71 (0.58 to 0.89)	0.002

General linearised modelling results, adjusted for hut position, sleeper pair and night

*CI* confidence intervals

### Relative humidity recordings

Relative humidity recordings from the elevated huts were similar to those recorded in the hut on the ground (Additional file 1: Table S3).

### Carbon dioxide measurements

Mean levels of carbon dioxide indoors were 32% higher than outdoors. Carbon dioxide levels indoors were high after the men entered the huts before gradually declining during the night with an uptick in concentration from 05.30 h to 07.00 h (Fig. 6). Huts on the ground had higher levels of carbon dioxide than elevated huts (Table 2).

Before midnight there was a 47% (95% CI 34–57%) decline in carbon dioxide at 2 m with air-permeable walls on the ground storey compared with the reference hut (Table 2). After midnight, there was a decrease of 19% (95% CI 11–27%) in the raised hut with an open ground storey and a 32% reduction (95% CI 23–56%) in the raised hut with air-permeable walls on the ground storey compared with the control hut.

### Serious adverse events

There were no adverse or serious adverse events during the study.

## Discussion

These findings establish how the permeability of the ground storey immediately below an inhabited hut elevated 2 m above the ground affects mosquito entry, indoor temperature and carbon dioxide levels. As found previously, the number of female *An. gambiae* mosquitoes collected in all raised huts was lower than the reference hut situated on the ground [11]. Elevated huts with no walls on the ground storey had 53% fewer mosquitoes than the comparator hut situated on the ground. This level of protection was slightly less than the 68% reported in the previous study, and this decrease in efficacy may be a result of adding a large wooden staircase to one side of each elevated hut in the present study, possibly guiding mosquitoes to an elevated hut. Mosquito hut entry was affected by presence of walls immediately below the elevated huts. Huts with a screened ground storey had 31% fewer house-entering mosquitoes and those with solid walls, 24% less than the hut at ground level. An analysis of mosquito counts restricted to elevated huts showed that huts with screened walls underneath had 63% more female *An. gambiae s.l.* and those with solid walls 45% more than the raised hut with no walls underneath. Similar levels of protection were observed with *Mansonia* spp. This was not the case for *Culex* spp., however, where there was no reduction in house entry in elevated huts compared to those on the ground. A similar finding was found in the previous study, so this is likely to be a true result reflecting the habits of these mosquitoes. This may be explained by the fact that, unlike *An. gambiae s.l.* and *Mansonia* spp. which fly low to the ground where there is some protection from the wind when host seeking, *Culex* spp. are routinely collected at higher altitudes [21], where they feed on birds at night. Linear regression analysis showed that as the numbers of *An. gambiae* increased, the mean number of *Mansonia* spp. also increased.

The major finding of this study is that screened or solid walls on the ground floor will reduce the entry of *An. gambiae s.l.* into an inhabited room on the second storey, but less effectively than in a raised hut that is open underneath. These findings are supported by a series of studies carried out in The Gambia in the 1970s by Gillies and Wilkes in the same area [14]. The experiment, conducted with a 6 m high circular fence made of mosquito screening and a human as bait in the centre, found that host-seeking mosquitoes flew up and over the fence towards the bait. Nonetheless, in a study in Tanzania [22], elevated houses with a screened second-storey and a screened or solid ground floor had 96% (95% CI 92–98) fewer mosquitoes than in the bedrooms of single storey houses. This finding supports the findings from the present work and suggests that elevating a house is an effective measure for preventing mosquito house entry.

The cues used by *An. gambiae* flying upwards to enter a hut are uncertain. The most parsimonious explanation is that when mosquitoes encounter a vertical barrier they are simply forced to fly upwards. This is not the case when *An. gambiae* enter houses, however, since they fly low to the ground across open country [12, 13], and when they approach a house, they do so at eaves (the gap between the top of the wall and the roof) level, not from below, following the line of the wall [23]. It is also important to appreciate that upwards flight and flight indoors are behaviours shown by endophagic mosquitoes, but not all mosquito species [24]. One alternative explanation is that mosquitoes are simply following odour plumes emanating from a raised hut. Visual cues may also contribute to house entry since Gillies and Wilkes demonstrated that mosquitoes were attracted to large shapes [25]. In the present experiment, the air-permeable and solid ground floor enclosures could have represented a large dark shape that guided mosquitoes towards each hut. Snow speculated that ‘at higher levels [above 2 m], where visual contact with the ground may be lost, direct orientation to the obstacles [in this case the elevated hut] could be increasingly important’ [26].

In the present study indoor temperature was several degrees higher than outdoor temperatures, but both decreased gradually during the night. There was no discernible reduction in indoor temperature with increasing height or in elevated huts whether the ground storey was walled or not.

Indoor carbon dioxide levels were 200 ppm higher in the huts compared with outdoor levels, providing a source of attraction for malaria mosquitoes especially on windless nights. The highest indoor carbon dioxide concentrations were at the beginning of the night, at 21.00 h but then declined slowly to the lowest level at 03.00 h. Indoor concentrations then started to rise after 05.30, increasing gradually until the sleepers left the huts at 07:00 h. The final increase might be due the natural circadian rhythm, with the body preparing to become active nearer dawn [27, 28] and an increase in physical activity immediately before leaving the huts. Carbon dioxide concentrations were higher in the hut located on the ground than in the raised huts through the night. This might be due to the presence of stronger winds affecting the raised huts. In contrast to temperature levels, carbon dioxide levels in huts were reduced by being elevated.

In recent decades, sub-Saharan Africa has experienced rapid urbanization, with migration from rural areas to the cities and the rising birth rate leading to 1.23 billion people living in African cities by 2050 [29]. The urgent need for new houses led to the uncontrolled growth of poorly-built constructions, commonly located in informal settlements. According to the World Bank the percentage of the population living in urban informal settlements has declined from 62% in 2000 to 55% in 2014 [30]. This, however, represents more than half of the urban population in sub-Saharan Africa. Recently, materials produced during the modern period like concrete blocks or corrugated metal roofing sheets are leading the house improvement phenomenon and market in sub-Saharan Africa [30]. They have played an important role in increasing of the percentage of houses built with finished materials from 32% (29–33%) in 2000 to 51% (49–54%) in 2015 [29]. As a consequence of globalization these materials are imported into sub-Saharan countries from China and India and have overtaken local construction markets and influenced design through massive constructions that serve as contemporary examples in the region [30]. With population growth and migration to cities, land becomes scarce, in that context the construction of multi-storey buildings is a solution to allocate more people in a plot of land. In peri-urban areas, like the ones created from rural–urban migration and urban expansion, where environmental conditions are more similar to rural than to urban contexts, vertical housing is a protective measure from malaria mosquitoes, but may not protect against nuisance biting by *Culex* mosquitoes [11]. The increase of vertical houses will also have implications in the pressure on basic services offered by the state, where electrical and water consumption will be more focalized and there will be a need for better infrastructure.

There are several limitations to the study. Firstly, the walls for the experimental huts were chosen because of their light weight and do not represent materials used commonly in local villages. This makes the huts’ thermal properties different from real houses. Secondly, the men entered the huts at 21:00 h and stayed until 07:00 h the next morning. This differs from normal behaviour where many people stay outside until midnight [20] and leave their houses for Fajr, morning prayer, before sunrise. Thirdly, the huts slept only two adult males, although rooms of four adults and children are more common in the local communities. Fourthly, when the huts were on the ground, there was an air gap under the floor which would have helped cool the hut more quickly than in a hut where the entire floor contacted the ground.

## Conclusion

These findings show that the number of malaria mosquitoes entering a hut is markedly reduced when the hut is raised 2 m above the ground and that if the space below the hut is walled in with either screening or solid walls, the protective effect is reduced. Ideally the ground floor should be left open when building multiple storey buildings. This research is likely to be particularly relevant to peri-urban areas of sub-Saharan Africa where new constructions are most likely to take place.

### Supplementary Information

**Additional file 1**: **Table S1**. Replicated Latin rectangle design for experimental huts heights. **Table S2. **Insect collection for all experimental nights. **Table S3**. Maximum and mean relative humidity levels in huts at different heights. **Figure S1. **Position of light trap (shown as x), data loggers (red) and sleepers. **Figure S2**. Experimental huts showing position of staircases. **Figure S3**. Wind direction and speed (10-1 ms^−1^) mosquito collection nights. A= 21.00 h to 23.59 h and B= 00.00 h to 06.59 h.

## Data Availability

Study protocols are available upon request. Anonymised data files are available on request and after approval of both the Scientific Coordinating Committee of the MRC Unit The Gambia at the London School of Hygiene and Tropical Medicine and the Joint Gambian Government/MRCG Ethics Committee.

## Abbreviations

CDC: CDC-Centers for Disease Control and Prevention
CI: Confidence intervals
ITN: Insecticide treated net
MRCG: Medical Research Council Unit The Gambia at the London School of Hygiene and Tropical Medicine
PCR: Polymerase chain reaction
UK: United Kingdom
USA: United States of America
