# Supplementary material for: The effect of physical barriers under a raised house on mosquito entry: an experimental study in rural Gambia
Source: Malar J. 2024 Apr 8;23:100. doi: 10.1186/s12936-024-04889-z (PMC11003187; doi:10.1186/s12936-024-04889-z)
Supplement: Supplementary file 1 — Additional file 1: Table S1. Replicated Latin rectangle design for experimental huts heights. Table S2. Insect collection for all experimental nights. Table S3. Maximum and mean relative humidity levels in huts at different heights. Figure S1. Position of light trap (shown as x), data loggers (red) and sleepers. Figure S2. Experimental huts showing position of staircases. Figure S3. Wind direction and speed (10-1 ms−1) mosquito collection nights. A= 21.00 h to 23.59 h and B= 00.00 h to 06.59 h. [file 12936_2024_4889_MOESM1_ESM.docx]

**Additional file 1**

**Table S1.** Replicated Latin rectangle design for experimental huts heights.

|  | Experimental Hut Typology | | | |
| --- | --- | --- | --- | --- |
| Session | Hut 1 | Hut 2 | Hut 3 | Hut 4 |
| 1 | 4 | 2 | 1 | 3 |
| 2 | 1 | 3 | 4 | 2 |
| 3 | 3 | 4 | 2 | 1 |
| 4 | 2 | 1 | 3 | 4 |
| 5 | 3 | 1 | 2 | 4 |
| 6 | 4 | 2 | 3 | 1 |
| 7 | 2 | 4 | 1 | 3 |
| 8 | 1 | 3 | 4 | 2 |
| 9 | 4 | 2 | 1 | 3 |

Typology 1: hut at ground level (control hut).

Typology 2: raised hut (2 m) with free ground storey.

Typology 3: raised hut (2m) with air-permeable walls in ground storey.

Typology 4: raised hut (2m) with solid walls in ground storey.

**Table S2.** Insect collection for all experimental nights.

|  | Hut typology | | | |  |
| --- | --- | --- | --- | --- | --- |
|  | Hut on the ground | Raised hut with solid walls on ground storey | Raised hut with air-permeable walls on ground storey | Raised hut with free ground storey |  |
|  |  |  |  |  |  |
| *An. gambiae* female | 1259 | 873 | 981 | 655 |  |
| *An. gambiae* male | 1 | 0 | 0 | 1 |  |
| *An. pharoensis* female | 1 | 0 | 0 | 3 |  |
| *An. pharoensis* male | 0 | 0 | 0 | 0 |  |
| *An. ziemani* female | 9 | 3 | 5 | 0 |  |
| *An. ziemani* male | 1 | 1 | 0 | 0 |  |
| *An. rufipes* female | 6 | 0 | 0 | 0 |  |
| *An. rufipes* male | 0 | 0 | 0 | 0 |  |
| *An. funestus* female | 11 | 6 | 4 | 2 |  |
| *An. funestus* male | 0 | 0 | 0 | 0 |  |
| *An. squamosus* female | 6 | 2 | 1 | 2 |  |
| *An. squamosus* male | 0 | 0 | 0 | 0 |  |
| *Culex quinquefsciatus* female | 610 | 588 | 645 | 598 |  |
| *Culex quinquefsciatus* male | 22 | 8 | 14 | 19 |  |
| *Cx thalassius* female | 34 | 58 | 52 | 45 |  |
| *Cx thalassius* male | 2 | 4 | 1 | 1 |  |
| *Mansonia* spp. female | 8535 | 4281 | 5021 | 4145 |  |
| *Mansonia* spp. male | 0 | 1 | 0 | 2 |  |
| *Aedes aegypti* female | 7 | 3 | 6 | 1 |  |
| *Aedes aegypti* male | 1 | 0 | 0 | 0 |  |

**Table S3**. Maximum and mean relative humidity levels in huts at different heights.

**
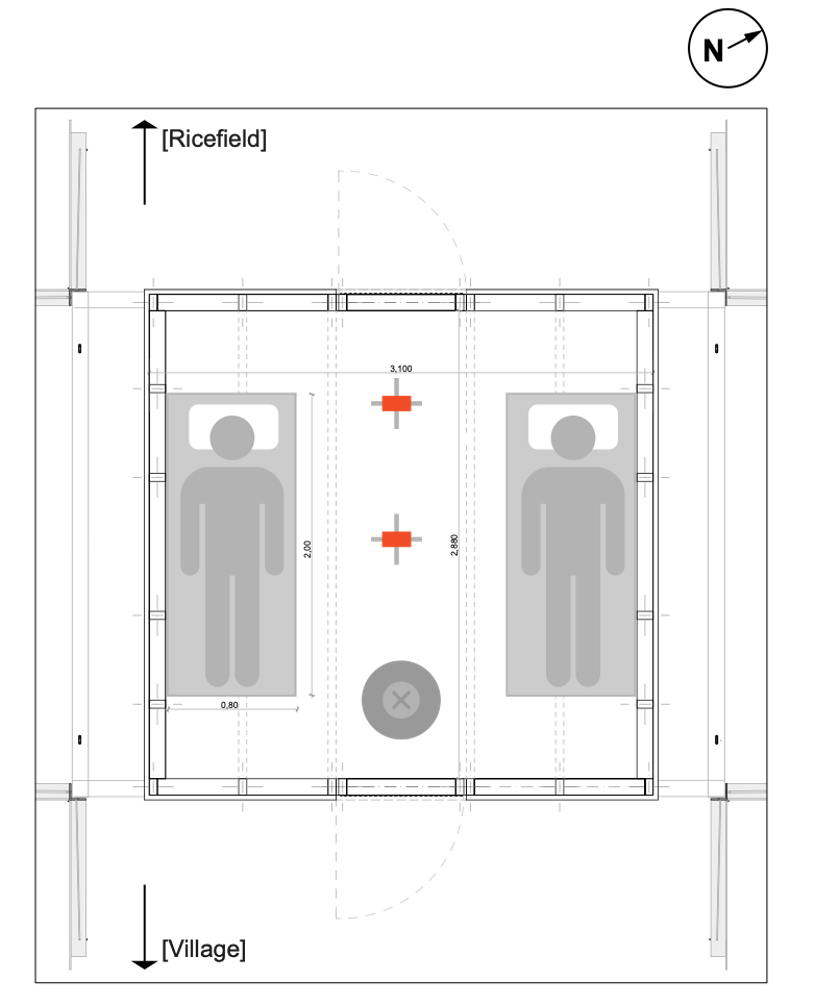
**

**Fig. S1.** Position of light trap (shown as x), data loggers (red) and sleepers.

*
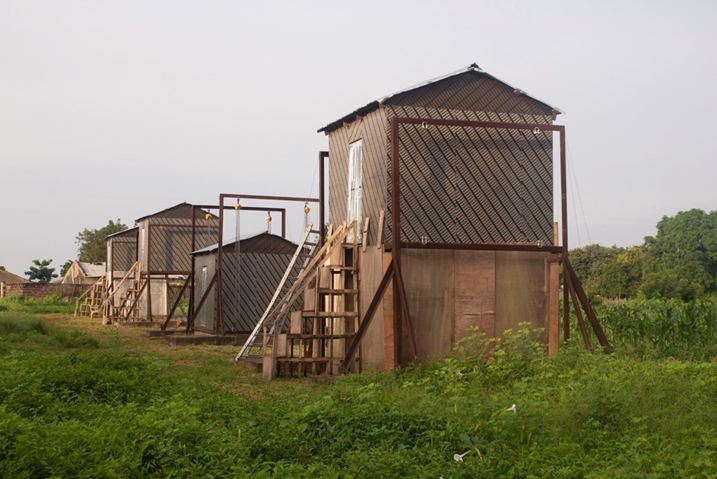
*

**Fig. S2.** Experimental huts showing position of staircases.

**Fig. S3.** Wind direction and speed (10^-1^ ms^-1^) mosquito collection nights. A= 21.00 h to 23.59 h and B= 00.00 h to 06.59 h.
